# Repurposing Brigatinib for the Treatment of Colorectal Cancer Based on Inhibition of ER-phagy

**DOI:** 10.7150/thno.36254

**Published:** 2019-07-09

**Authors:** Zhe Zhang, Wei Gao, Li Zhou, Yan Chen, Siyuan Qin, Lu Zhang, Jiayang Liu, Yujia He, Yunlong Lei, Hai-Ning Chen, Junhong Han, Zong-Guang Zhou, Edouard C. Nice, Changlong Li, Canhua Huang, Xiawei Wei

**Affiliations:** 1 State Key Laboratory of Biotherapy and Cancer Center, West China Hospital, and West China School of Basic Medical Sciences & Forensic Medicine, Sichuan University, and Collaborative Innovation Center for Biotherapy, Chengdu, 610041, P. R. China; 2 West China School of Basic Medical Sciences & Forensic Medicine, Sichuan University, Chengdu, 610041, P. R. China; 3 Department of Biochemistry and Molecular Biology, and Molecular Medicine and Cancer Research Center, Chongqing Medical University, Chongqing 400016, P. R. China; 4 Department of Gastrointestinal Surgery, State Key Laboratory of Biotherapy and Cancer Center, West China Hospital, Sichuan University, and Collaborative Innovation Center for Biotherapy, Chengdu, 610041, P. R. China; 5 Department of Biochemistry and Molecular Biology, Monash University, Clayton, Victoria, Australia; 6 Laboratory of Aging Research and Cancer Drug Target, State Key Laboratory of Biotherapy and Cancer Center, National Clinical Research Center for Geriatrics, West China Hospital, Sichuan University, Chengdu, 610041, P. R. China

**Keywords:** Brigatinib, ALK independence, ER stress, ER-phagy, Colorectal cancer

## Abstract

**Rationale**: The sustained and severe endoplasmic reticulum (ER) stress in cancer cells may contribute to apoptotic cell death, thus representing a potential target for cancer therapy. Brigatinib is an anaplastic lymphoma kinase (ALK) inhibitor approved for the treatment of ALK-positive non-small-cell lung cancer (NSCLC). However, it remains unclear if brigatinib has alternative model of action to exert antitumor effect in ALK-negative cancers.

**Methods**: ALK-positive NSCLC cells and various human ALK-negative cancer cells, especially human colorectal cancer (CRC) cells were used to examine the tumor suppression effect of brigatinib alone or in combination with autophagy inhibitors* in vitro* and *in vivo*. A variety of biochemical assays were conducted to elucidate the underlying mechanisms of brigatinib in CRC cells.

**Results**: Here, we show the significant anti-cancer effect of brigatinib in CRC through induction of apoptosis by sustained ER stress. Mechanistically, brigatinib induces ER stress via promoting the interaction between ubiquitin-specific peptidase 5 (USP5), a deubiquitinase, and oxysterol-binding protein-related protein 8 (ORP8), leading to ORP8 deubiquitination, accumulation and growth inhibition. Furthermore, we find that brigatinib-mediated ER stress simultaneously induces autophagic response via ER-phagy that acts as a protective mechanism to relieve excessive ER stress. As such, combination of brigatinib with autophagy inhibitors significantly enhances the anti-CRC effect of brigatinib both *in vitro* and* in vivo*, supporting the repurposing of brigatinib in CRC, independently of ALK.

**Conclusion**: The unearthed new molecular action of brigatinib suggests that therapeutic modulation of ER stress and autophagy might represent a valid strategy to treat CRC and perhaps other ALK-negative cancers.

## Introduction

Endoplasmic reticulum (ER) stress and unfolded protein response (UPR) have attracted much attention as therapeutic targets for cancer treatment in recent years 1, 2. The ER is responsible for the correct folding and posttranslational modifications of proteins, calcium storage, detoxification of chemical compounds, as well as lipid synthesis 3, 4. Perturbation of ER homeostasis, usually leading to ER stress, provokes UPR to cope with the resultant stress 5. PERK, IRE1α and ATF6α, the major sensors of ER stress and markers of UPR, can promote the transcription of related genes responsible for maintaining ER homeostasis, but under certain conditions may prolong ER stress and eventually morph into pro-apoptotic pathways 5-7. Several reports have shown that the induction of apoptosis by ER stress is responsible for cancer cell death in various cancers, suggesting that UPR activation can be potentially tumor-suppressive, especially when ER stress is intensive and persistent 1, 8.

Macroautophagy is responsible for the degradation and recycling of macromolecules and organelles to maintain cellular homeostasis and can be activated in stress conditions, including ER stress 3, 9, 10. It has been well established that autophagosomes can recognize specific substrates including organelles 11. ER-phagy, a type of cargo-specific autophagy degrading ER, has commonly been recognized as a form of basal quality control mechanism to counterbalance ER expansion during UPR 11, 12. The selective autophagy is mediated by autophagy receptors that recruit the designated targets to autophagosomal membranes by binding to LC3/GABARAP 13, 14. Family with sequence similarity 134 member B (FAM134B) protein, one of the ER-resident receptors, binds to LC3/GABARAP via its LC3-interacting region (LIR), and facilitates ER degradation by ER-phagy 15. Growing evidence has indicated that ER-phagy is a clearance outcome of ER stress in certain conditions 9, 16. However, whether ER-phagy is involved in autophagy-mediated therapeutic response to anti-cancer drugs is not clear.

Repurposing existing drugs offers a cost-effective and time-saving approach for drug discovery in cancer therapy. Drug repurposing is based on two concepts: on-target repurposing (finding new therapeutic indications based on relevant mechanisms) and off-target repurposing (identifying new targets for known compounds) 17. Brigatinib received its first global approval in 2017 due to the robust efficacy in patients with anaplastic lymphoma kinase (ALK)-positive non-small-cell lung cancer (NSCLC) 18, 19. The growth inhibition effect of brigatinib was also evident in other ALK-positive tumors, such as neuroblastoma and anaplastic large cell lymphoma 20, 21. However, few studies regarding the off-target effect of brigatinib in tumor therapy have been reported.

In this study, we found an ALK-independent anti-cancer mechanism of brigatinib in colorectal cancer (CRC). Brigatinib stimulates ER stress by USP5-mediated ORP8 stabilization to promote apoptotic cell death of CRC cells both *in vitro* and *in vivo*. Meanwhile, ER-phagy is stimulated to relieve ER stress in brigatinib-treated CRC cells. Collectively, our results provide novel insights into the molecular basis for the anti-cancer effect of brigatinib and demonstrate the potential of brigatinib for the treatment of ALK-negative cancer.

## Materials and Methods

### Cell culture

Cell culture was performed at 37°C in a humidified atmosphere containing 5% CO_2_. Human CRC cell lines (DLD-1, HCT116, HT29, RKO and SW620), human colon mucosal epithelial cell line NCM460, human NSCLC cell line A549, human HCC cell line Hep3B and human prostate cancer cell line Du145 were purchased from ATCC (Manassas, VA, USA). NSCLC cell lines H3122 and H2228 were kindly provided by Prof. Yong Peng (State Key Laboratory of Biotherapy, Sichuan University). Du145, H3122 and H2228 cell lines were cultured in RPMI Medium 1640, other cell lines were cultured in high glucose Dulbecco's Modified Eagle Medium (DMEM, Gibco) and were supplemented with 10% FBS (Biowest), 100 U/mL penicillin, 100 U/mL streptomycin (Hyclone).

### Antibodies and reagents

All commercial antibodies used in this study were obtained from the following suppliers: BiP (sc-376768), CHOP (sc-56107), PERK (sc-377400), p-PERK (sc-32577), IRE1α (sc-390960), ORP8 (sc-134409), USP5 (sc-390943), JNK (sc-7345), p-JNK (sc-81502), horseradish peroxidase-conjugated anti-rabbit secondary antibody (sc-2004) and horseradish peroxidase-conjugated anti-mouse secondary antibody (sc-2005) were obtained from Santa Cruz Biotechnology; p-IRE1α (ab48187) and HA (ab9110) were obtained from Abcam; LC3 (NB100-2220) was obtained from Novus; FAM134B (PA5-64943), goat anti-mouse Alexa Fluor 488 (A28175), goat anti-rabbit Alexa Fluor 488 (A27034), goat anti-mouse Alexa Fluor 594 (A21044) and goat anti-rabbit Alexa Fluor 594 (A32740) were obtained from Invitrogen. PARP (9532), Cleaved PARP (5625), Caspase-3 (9662), Cleaved caspase-3 (9664), ATG5 (12994S), ATG7 (8558S), Beclin1 (3738), ATG14 (96752) and Bcl-2 (15071) were obtained from Cell Signaling Technology.

The following commercial chemical reagents were obtained from the following suppliers: brigatinib (HY-12857), Z-VAD(OMe)-FMK (HY-16658), 3-Methyladenine (HY-19312), bafilomycin A1 (HY-100558), 5-FU (HY-90006) and Cycloheximide (HY-12320) were obtained from MedChemExpress; 4-PBA (1716-12-7) and chloroquine diphosphate salt (C6628) were obtained from MilliporeSigma. All chemicals were handled in accordance with the supplier's recommendations.

### Detection of cell growth

The growth of brigatinib-treated cells was determined using the MTT assay. Cells were plated in 96-well plates at 5,000 cells per well and subjected to different treatments. The detailed procedure has been previously described 22. For the colony formation assay, cells were cultured in 24-well plates with different treatments. The colonies were counterstained with Giemsa after 2 weeks, then washed 3 times by PBS. The visible colonies were recorded using a Molecular Imager Gel Do XR+ System (BIO-RAD) and counted using Image J software (NIH). EdU Cell Proliferation Assay Kit (Ribobio, Guangzhou, China) was used to perform the 5-ethynyl-20-deoxyuridine (EdU) labeling assay. The detailed procedure has been previously described 23.

### Lactate dehydrogenase release assay

Lactate dehydrogenase release was used to detect cytotoxicity following different treatments, using a lactate dehydrogenase (LDH) test kit (Beyotime Biotechnology, Nanjing, China). Studies were performed according to the supplier's instruction.

### TUNEL assays

The DeadEndTM Fluorometric TUNEL system (Promega) was used to detect apoptotic cells according to the manufacturer's instructions. The apoptotic and non-apoptotic signals were recorded using a fluorescent microscope and the percentage of cells with DNA nick end-labelling evaluated.

### Flow cytometry

The apoptotic ratio was determined with a FITC-Annexin V Apoptosis Detection kit (KeyGEN BioTECH, KGA108). In brief, cells were harvested and washed once with PBS, and then resuspended in PI/Annexin-V solution for apoptosis analysis. The detailed procedures were performed according to the corresponding manufacturer's instructions. At least 10,000 live cells were analyzed on a FACSCalibur flow cytometer (Becton Dickinson). Data were analyzed by using FlowJo software.

### Immunoblotting and immunoprecipitation

Cells were rinsed in precooled PBS and lysed in RIPA lysis buffer (150 mM Tris-HCl, pH 7.5, 150 mM NaCl, 1% NP-40 and protease inhibitors). Equal amounts of soluble proteins (15-30 μg) were used for immunoblotting analysis. For immunoprecipitations, cells were lysed with IP lysis buffer (20 mM Tris,137 mM NaCl, 10% glycerol, 1% NP-40 and 2 mM MEDTA, pH=7.5). The whole-cell lysates were subjected to immunoprecipitation overnight at 4°C with 1 μg of the indicated antibodies, and the immunoprecipitated protein was pulled down with Protein A agarose beads (40 μl; GE Healthcare, Chicago, IL, USA) for 4 h. Proteins were visualized with Immobilon Western HRP Substrate (Millipore, WBKLS0050). The images were obtained using a ChemiScope 6000 Touch (Clinx, Shanghai, China).

### Immunofluorescence

Cells were cultured on glass coverslips overnight and treated with different agents in 24-well plates (5×10^3^ cells/well). After being fixed with 4% paraformaldehyde (Sigma) for 30 minutes, cells were permeabilized with 0.4% Triton X-100 followed by blocking with 5% fetal bovine serum after being washed 3 times with PBS. The treated cells were then incubated with the indicated primary antibodies and Alexa Flour secondary antibodies. Lastly, cells were visualized using a Zeiss LSM 510 confocal microscope. For ER labelling, live cells were previously stained with 100 nM ER-Tracker Blue for 30 minutes at 37℃.

### Immunohistochemistry

Immunohistochemical analysis was performed as previously described 22. All samples were visualized under a Leica DM 2000 microscope. Quantitative scoring analysis was performed by multiplying the percentage of staining-positive cells area (A) by the immunostaining intensity (B: 0, negative; 1, weakly positive; 2, positive; 3, strongly positive). The final score for each slide was calculated as A×B.

### siRNA transfection

All siRNAs were synthesized by GenePharma (Shanghai, China) and were transfected with Lipofectamine 3000 reagent (Thermo Fisher Scientific) for 48 h according to the manufacturer's protocol. The sequences of siRNA involved in this study were as follows: si*ORP8*: 5'-GAGUGGUCUUGCAAAUUAU-3', si*ATG5*: 5'-GCAACUCUGGAUGGGAUUG-3', si*BECN1*: 5'-CAGUUUGGCACAAUCAAUATT-3', si*ATG7*: 5'-CAGUGGAUCUAAAUCUCAAACUGAU-3', si*IRE1α*: 5'-GGACGUGAGCGACAGAAUA-3', si*PERK*: 5'-CAACAAGAAUAUCCGCAAA-3', si*FAM134B*: 5'-GAGGUAUCCUGGACUGAUA-3'.

### Plasmids

The human FAM134B coding region with C-terminal HA tag was cloned using PCR and was ligated into the pcDNA3.1(+) vector. The origin PCR primers for wild type FAM134B sequence were as follows: Sense primer: 5'-CCCGGATCCATGGCGAGCCCG-3'; Anti-sense primer: 5'-CCGCTCGAGTTAATGGCCTCCCAG-3'. The PCR primers for LIR-motif mutant type FAM134B sequence were as follows: Sense primer: 5'-ACTGAAGAAGGTGCTGCCGCTGCAGCAGCTGACCAGTCAGAG-3'; Anti-sense primer: 5'-GCTGCTGCAGCGGCAGCACCTTCTTCAGTGTCTGTGTCCTC-3'.

### Tumor xenograft model

Female nude mice (BALB/c, non-fertile, and 18-20 g each) at 6 weeks of age were purchased from HFK Bioscience (Beijing, China). For the subcutaneous xenograft model, DLD-1 cells (1×10^7^ cells/mouse) were suspended in PBS and injected subcutaneously into mice. When the tumor volume reached ~100 mm^3^, the mice were randomized into vehicle and treatment groups. The mice were treated once daily by oral gavage (control, 75% physiologic saline, 25% Medicinal alcohol; brigatinib, 75 mg/kg/day; CQ, 25 mg/kg/day; brigatinib + CQ, brigatinib 75 mg/kg/day + CQ 25 mg/kg/day). Tumor volumes were measured each day and evaluated according to the following formula: tumor volume (mm^3^) = (length×width^2^)/2. When significant differences between each group were obtained, the mice were euthanized and tumor tissues isolated and fixed immediately in 10% formalin. All animal experiments were approved by the Institutional Animal Care and Treatment Committee of Sichuan University.

### Statistical Analysis

All statistical analysis and graphics were performed using GraphPad 6 software (GraphPad, La Jolla, CA, USA). One-way ANOVA or Student's t test was used to analyze statistical differences. A value of *P* < 0.05 was considered as statistically significant. Error bars indicated SEM unless otherwise indicated.

## Results

### Brigatinib inhibits the growth of CRC cells

To examine whether brigatinib exhibits a growth inhibition effect in ALK-negative cancer cells, ALK-positive NSCLC cell line (H3122 and H2228) and various ALK-negative cancer cells lines (A549, Hep3B, Du145, HCT116) were treated with brigatinib. As shown in Figure S1A, brigatinib treatment significantly reduced the growth of H3122 and H2228 cells in a dose-dependent manner. Interestingly, brigatinib also showed obvious anti-neoplastic activity in several ALK-negative cancer cell lines (Figure S1B), suggesting the presence of an ALK-independent anti-cancer mechanism for brigatinib.

**Figure 1:**
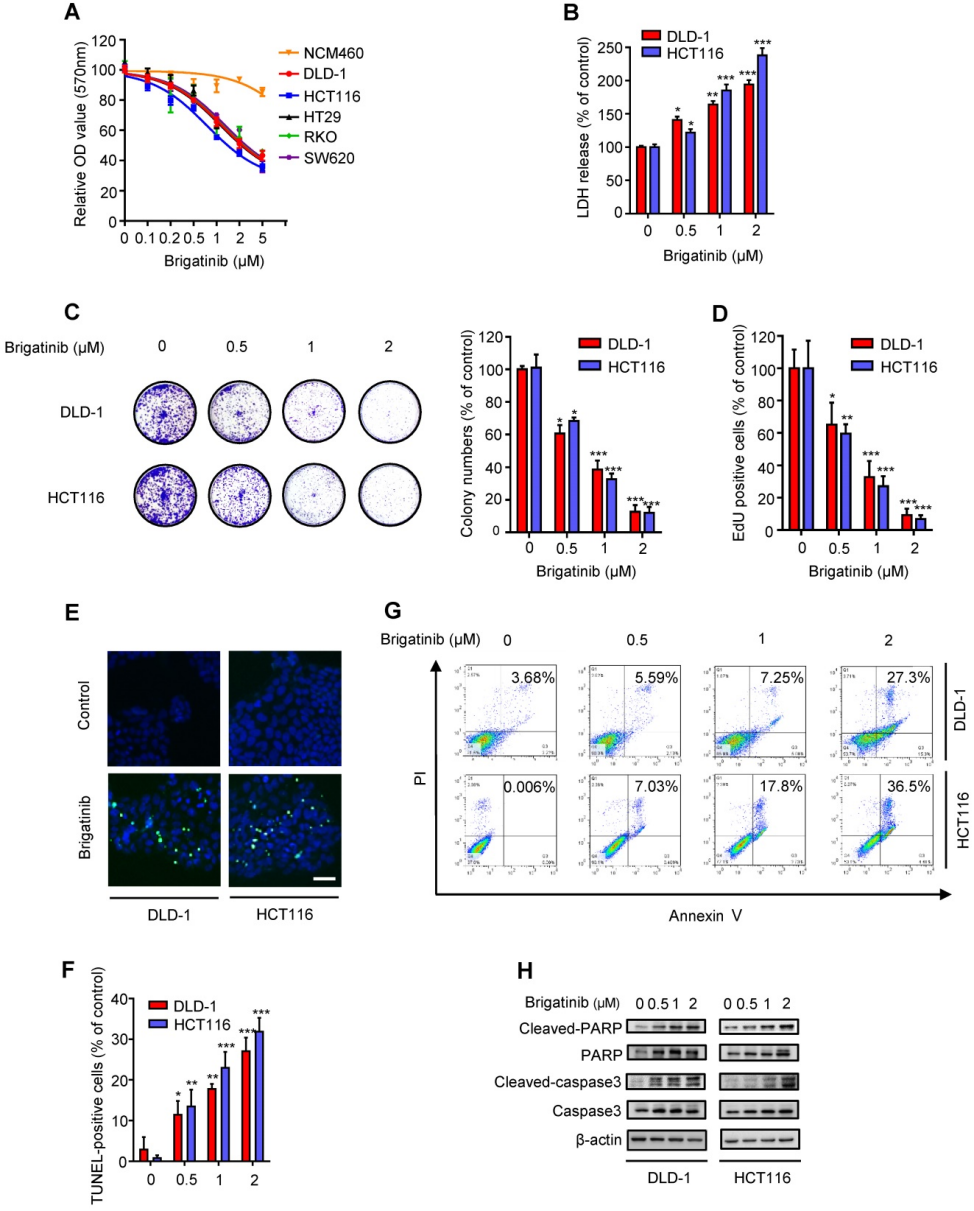
** Brigatinib inhibits the growth of CRC cells. A,** Cell growth of various CRC cell lines treated with the indicated concentrations of brigatinib for 24 hours. **B,** LDH release assay of DLD-1 and HCT116 cells treated with the indicated concentrations of brigatinib for 24 hours. *, *P* < 0.05; **, *P* < 0.01; ***, *P* < 0.001. **C,** Colony formation assay of CRC cells treated with the indicated concentrations of brigatinib. Representative images (Left) and quantification of colonies (Right) were shown. *, *P* < 0.05; ***, *P* < 0.001. **D,** EdU labeling assay. Cells were treated as in (B). *, *P* < 0.05; **, *P* < 0.01; ***, *P* < 0.001. **E-F,** TUNEL assay in cells treated as in (B). Representative images (E) and quantification of TUNEL-positive cells (F) were shown. Scale bar, 50 μm. *, *P* < 0.05; **, *P* < 0.01; ***, *P* < 0.001. **G,** Flow cytometric analysis of apoptosis in cells treated as in (B). **H,** Immunoblotting of total and cleaved PARP or caspase 3 in CRC cells treated with the indicated concentrations of brigatinib for 24 hours.

To ascertain the anti-cancer effect of brigatinib against CRC cell lines, cell growth was assessed following brigatinib treatment in a variety of CRC cell lines (DLD-1, HCT116, HT29, RKO, SW620) and a human colon mucosal epithelial cell line, NCM460. As expected, all tested CRC cells were sensitive to brigatinib at 2 μM for 24 hours, whereas NCM460 cells demonstrated higher tolerance to brigatinib (Figure 1A). Moreover, LDH release assay revealed that brigatinib treatment exhibited marked cytotoxicity in CRC cells (Figure 1B). Consistently, the proliferation of CRC cells was significantly inhibited under brigatinib treatment, as evidenced by reduced colony formation (Figure 1C) and EdU incorporation (Figure 1D) in brigatinib-treated CRC cells. Together, these results indicate that brigatinib demonstrates a considerable anti-cancer effect in CRC cells *in vitro*.

To examine whether apoptosis was associated with the anti-cancer effect of brigatinib, we evaluated the apoptotic ratio using both TUNEL and flow cytometry assays. As shown as Figure 1E-G, brigatinib treatment for 24 hours showed an obvious effect on apoptosis induction in CRC cells. Increased cleaved-caspase 3 and cleaved-PARP were also observed in brigatinib-treated CRC cells (Figure 1H). Of note, treatment with apoptosis inhibitor zVAD-FMk (zVAD) inhibited brigatinib-induced cytotoxicity in CRC cells (Figure S2). Taken together, our data demonstrate that brigatinib inhibits CRC growth by inducing apoptosis.

### Brigatinib induces apoptosis by activating ER stress in CRC cells

**Figure 2:**
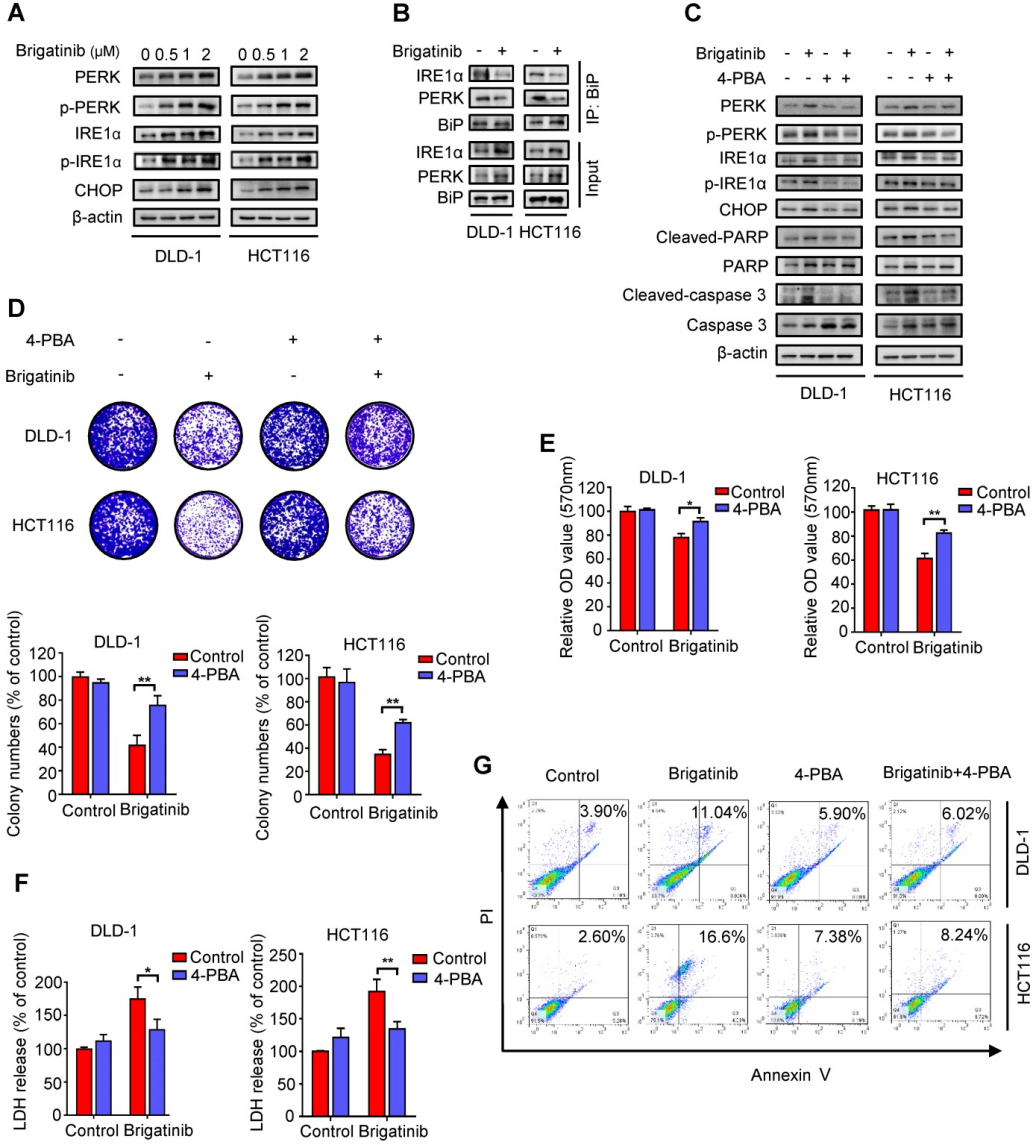
** Brigatinib induces apoptosis by activating ER stress in CRC cells. A,** Immunoblotting of total and phosphorylated PERK, IRE1α and CHOP in CRC cells treated with the indicated concentrations of brigatinib for 24 hours. **B,** Interaction among BiP, PERK, and IRE1α was determined by coimmunoprecipitation assay. **C,** Immunoblotting of total and phosphorylated PERK, IRE1α and CHOP as well as total and cleaved PARP or caspase 3 in CRC cells treated with or without 1 μM brigatinib in the presence or absence of 2 mM 4-phenylbutyrate (4-PBA) for 24 hours. **D,** Colony formation assay of CRC cells treated with or without 1 μM brigatinib in the presence or absence of 2 mM 4-PBA. Representative images (Top) and Quantification of colonies (Bottom) were shown. **, *P* < 0.01. **E-G**, Cell growth assay (E), LDH release assay (F) and flow cytometric analysis of apoptosis (G) in CRC cells treated as (D). *, *P* < 0.05; **, *P* < 0.01.

Growing evidence has indicated that ER stress is closely linked to apoptosis induction 2, 8, 24. To ascertain whether ER stress was activated by brigatinib in CRC cells, we examined the levels of classic ER stress markers, including PERK, p-PERK, IRE1α, p-IRE1α and CHOP. We observed increased levels of these ER stress markers upon brigatinib treatment (Figure 2A), indicating activation of ER stress. Meanwhile, we found that brigatinib treatment significantly reduced the interaction of BiP with PERK or IRE1α (Figure 2B), further supporting the stimulation of ER stress in CRC cells. To evaluate whether brigatinib-induced ER stress led to apoptosis induction, CRC cells were treated with brigatinib combined with an ER stress inhibitor, 4-phenylbutyrate (4-PBA). As shown in Figure 2C, 4-PBA markedly decreased brigatinib-induced overexpression of the tested ER stress markers. An increase in cell proliferation was also observed in brigatinib-treated CRC cells in combination with 4-PBA (Figure 2D-E). In addition, LDH release also revealed that 4-PBA counteracted brigatinib-induced cytotoxicity (Figure 2F). Consistently, the ratio of apoptotic cells was also reduced by combinatorial treatment of 4-PBA with brigatinib (Figure 2G). Taken together, these results indicate that ER stress contributes to brigatinib-induced apoptosis in CRC cells.

Interestingly, brigatinib treatment promoted classic ER signaling protein markers (including PERK, p-PERK, IRE1α, p-IRE1α and CHOP) in ALK-negative cancer cell lines (A549, Hep3B and Du145) (Figure S3A), but not in ALK-positive cancer cells (H3122 and H2228) (Figure S3B). In line with this, 4-PBA restored brigatinib-stimulated ER stress in ALK-negative cancer cell lines (A549, Hep3B and Du145). In addition, zVAD partially rescued brigatinib-induced growth suppression in both ALK-negative (A549, Hep3B and Du145) and ALK-positive (H3122 and H2228) cancer cell lines, whereas 4-PBA compromised brigatinib-induced growth inhibition in ALK-negative cancer cells with no obvious change in ALK-positive cancer cells (Figure S3C). These results demonstrate that brigatinib inhibits cell growth, at least in part, by ER stress-induced apoptosis in ALK-negative cancer cells.

### USP5-mediated ORP8 accumulation contributes to brigatinib-induced ER stress in CRC cells

Next, we set out to investigate the mechanism underlying brigatinib-induced ER stress. Phospholipids, the main component of biological membranes, play a crucial role in the functional and structural aspects of ER 25, 26. We hypothesized that disordered processing of phospholipids transportation was a potential inducer for ER stress. Oxysterol-binding protein (OSBP)-related protein 5/8 (ORP5/8) were the only two lipid transfer proteins anchored to ER membranes 27, 28 and ORP8 expression was closely related to tumor cell apoptosis 29-31.

**Figure 3:**
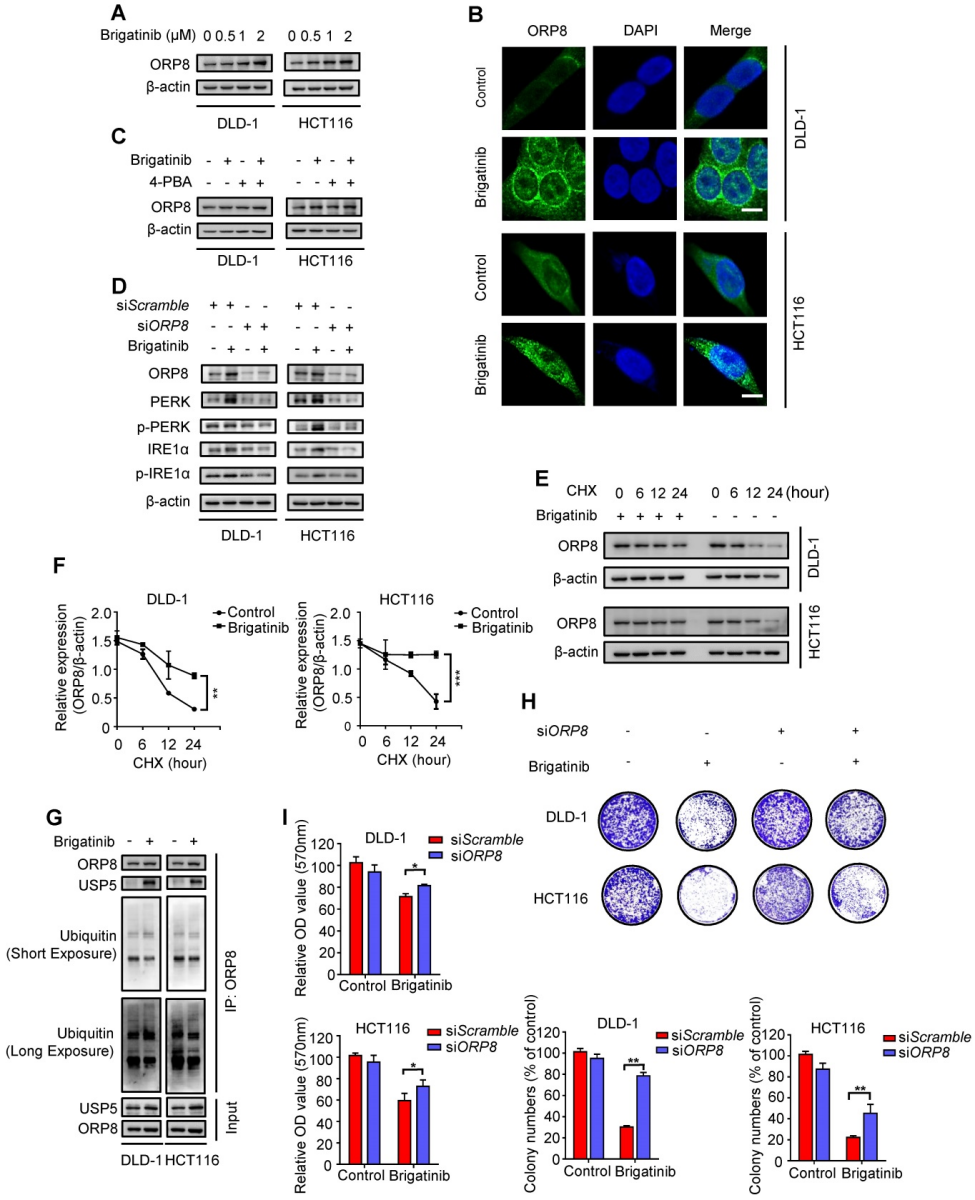
** USP5-mediated ORP8 accumulation contributes to brigatinib-induced ER stress in CRC cells**. **A,** Immunoblotting of ORP8 in CRC cells treated with the indicated concentrations of brigatinib for 24 hours. **B,** Immunofluorescence analysis of ORP8 in CRC cells treated with 1 μM brigatinib for 12 hours. Scale bar, 10 μm. **C,** Immunoblotting of ORP8 in CRC cells treated with or without 1 μM brigatinib in the presence or absence of 2 mM 4-PBA for 24 hours. **D,** Immunoblotting of total and phosphorylated PERK, IRE1α in CRC cells transfected with si*ORP8* or si*Scramble* followed by treatment with or without 1 μM brigatinib for 24 hours. **E-F,** Immunoblotting (E) of ORP8 in CRC cells pretreated with 1 μM brigatinib for 12 hours followed by exposure to 50 μg CHX combined with or without 1 μM brigatinib for the indicated time. Quantification of relative ORP8 expression (F) was shown. **, *P* < 0.01; ***, *P* < 0.001. **G,** Coimmunoprecipitation showing the interaction among ORP8, USP5 and ubiquitin after brigatinib treatment. **H,** Colony formation assay of CRC cells transfected with si*ORP8* or si*Scramble* followed by treatment with or without 1 μM brigatinib. Representative images (Top) and quantification of colonies (Bottom) were shown. **, *P* < 0.01. **I,** Cell growth of CRC cells transfected with si*ORP8* or si*Scramble* followed by treatment with or without 1 μM brigatinib for 24 hours. *, *P* < 0.05.

To ascertain our hypothesis, we examined ORP8 expression following brigatinib treatment and found increased ORP8 expression in brigatinib-treated CRC cells (Figure 3A). Brigatinib-induced ORP8 up-regulation was further confirmed by immunofluorescence (Figure 3B). Meanwhile, 4-PBA had no obvious effect on ORP8 expression in brigatinib-treated CRC cells (Figure 3C). However, knockdown of ORP8 by si*ORP8* significantly countered brigatinib-induced up-regulation of ER stress markers and activation of caspase (Figure 3D and Figure S4). These data suggest that ER stress is a downstream molecular event of brigatinib-induced ORP8 expression in CRC cells.

We next asked whether the overexpression of ORP8 in brigatinib-treated CRC cells was due to reduced proteasomal degradation. As expected, cycloheximide (CHX, a protein synthesis inhibitor) time-course analysis showed that brigatinib reduced the rate of ORP8 degradation (Figure 3E-F). We then investigated several deubiquitylases, and found an increase in the interaction between USP5 and ORP8 under brigatinib treatment, along with decreased ORP8 ubiquitination (Figure 3G). We next examined the role of ORP8 in brigatinib-induced growth suppression in CRC cells. As shown in Figure 3H-I, siRNA mediated *ORP8* knockdown could significantly decrease growth suppression in brigatinib-treated CRC cells, suggesting that ORP8 contributes to brigatinib-induced cytotoxicity. Taken together, these data suggest that increased interaction between USP5 and ORP8 facilitates the accumulation of ORP8 via USP5-mediated deubiquitination, leading to aggravation of ER stress in brigatinib-treated CRC cells.

### Brigatinib induces autophagy via ER stress in CRC cells

**Figure 4:**
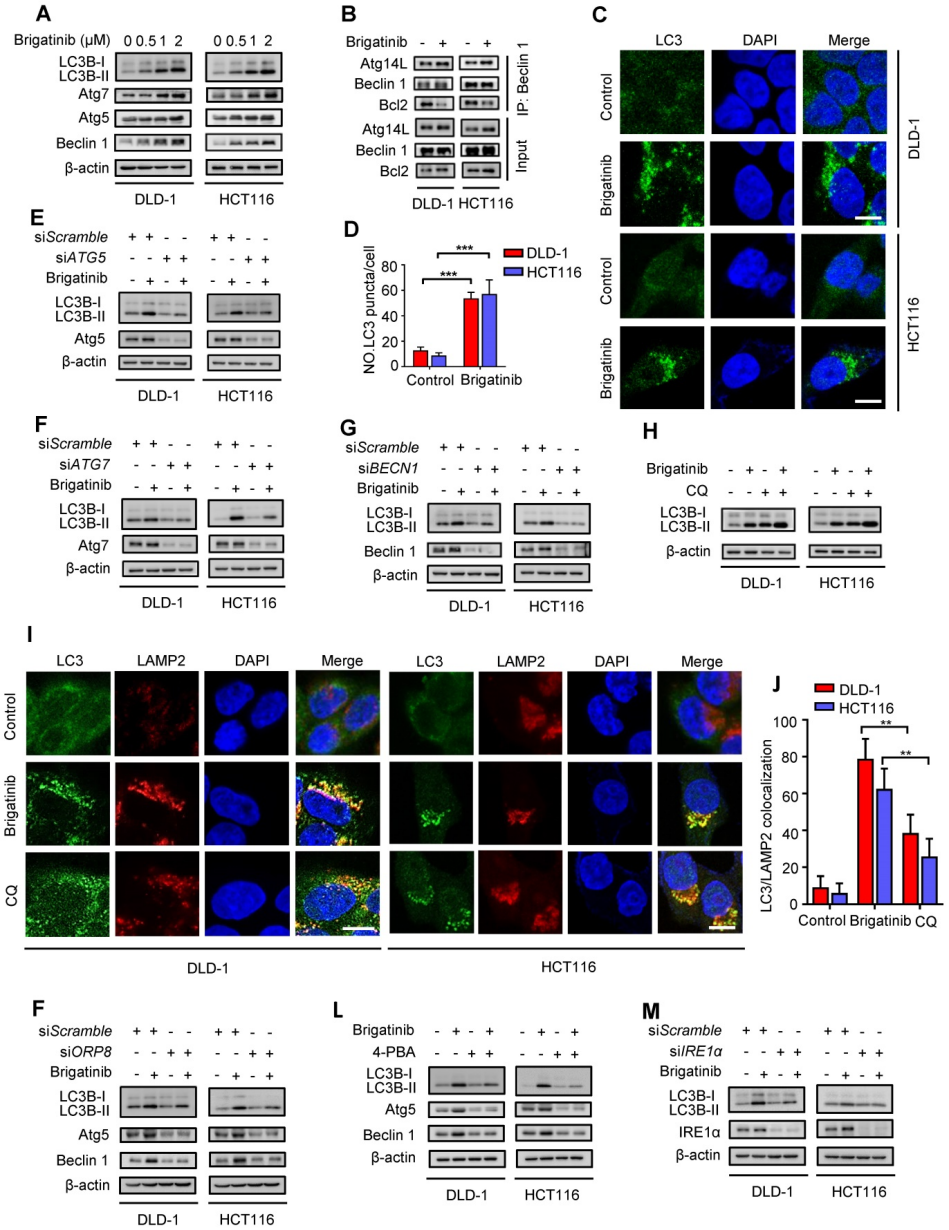
** Brigatinib activates autophagy via ER stress-signaling pathway in CRC cells. A,** Immunoblotting of LC3B, Atg5, Atg7, and Beclin 1 in CRC cells treated with the indicated concentrations of brigatinib for 24 hours. **B,** Interaction among Beclin 1, Bcl-2 and Atg14L was determined by coimmunoprecipitation assay.** C-D**, Immunofluorescence analysis (C) of LC3B in CRC cells treated with or without 1 µM brigatinib for 12 hours. The number of LC3 puncta (D) was shown. ***, *P* < 0.001. Scale bar, 10 μm. **E,** Immunoblotting of LC3B in CRC cells transfected with si*ATG5* or si*Scramble* followed by treatment with or without 1 μM brigatinib for 24 hours.** F,** Immunoblotting of LC3B in CRC cells transfected with si*ATG7* or si*Scramble* followed by treatment with or without 1 μM brigatinib for 24 hours. **G,** Immunoblotting of LC3B in CRC cells transfected with si*BECN1* or si*Scramble* followed by treatment with or without 1 μM brigatinib for 24 hours. **H,** Immunoblotting of LC3B in CRC cells treated with or without 1 μM brigatinib in the presence or absence of 10 μM chloroquine (CQ) for 24 hours. **I-J,** Immunofluorescence analysis (I) of colocalized LC3B and LAMP2 in CRC cells treated with or without 1 µM brigatinib for 12 hours. CQ group was negative control. The number of colocalized or non-colocalized LC3B and LAMP2 (J) was quantified. *, *P* < 0.05. **, *P* < 0.01. Scale bar, 10 μm.** K,** Immunoblotting of LC3B, Atg5 and Beclin 1 in CRC cells transfected with si*ORP8* or si*Scramble* followed by treatment with or without 1 μM brigatinib for 24 hours.** L,** Immunoblotting of LC3B, Atg5 and Beclin 1 in CRC cells treated with or without 1 μM brigatinib in the presence or absence of 2 mM 4-PBA for 24 hours.** M,** Immunoblotting of LC3B in CRC cells transfected with si*IRE1α* or si*Scramble* followed by treatment with or without 1 μM brigatinib for 24 hours.

Autophagy is an evolutionarily conserved mechanism for maintaining cellular homeostasis 3, 32. Growing evidence indicates that activation of UPR induced by ER stress promotes autophagy to coordinate ER homeostasis 33-35. To clarify whether brigatinib induced autophagy in CRC cells, we investigated the conversion of LC3B-I to lipidated LC3B-II (an established autophagosome marker) and the levels of autophagy-related proteins (Atg5, Atg7, Beclin 1). As shown in Figure 4A, brigatinib treatment resulted in marked autophagy induction, as evidenced by increased LC3B-II conversion and levels of Atg5, Atg7 and Beclin 1 in a dose dependent manner. Brigatinib treatment also increased the interaction of Beclin 1 with Atg14L and decreased the binding of Beclin 1 with Bcl-2 (Figure 4B). In addition, the formation of endogenous LC3B and GFP-LC3 puncta was dramatically increased in brigatinib-treated CRC cells (Figure 4C-D and Figure S5A-B). siRNA-mediated *ATG5*, *ATG7* or *BECN1* silencing prominently decreased LC3B lipidation in brigatinib-treated cells (Figure 4E-G). Thus, these data suggest that brigatinib promotes the initiation process of autophagy in CRC cells.

We next evaluated whether brigatinib promoted the autophagy flux in CRC cells. Combinatorial treatment of brigatinib with autolysosome inhibitor (Chloroquine, CQ) resulted in a further increase in LC3B lipidation (Figure 4H). Moreover, we observed that brigatinib treatment induced obvious colocalization of LC3 with LAMP2 (lysosome marker) (Figure 4I-J), suggesting the fusion of the autophagosome with lysosome. Additionally, using a tandem mRFP-GFP tagged LC3 construct, we found that brigatinib-treated CRC cells displayed more autolysosomes (GFP^-^RFP^+^ signal) than autophagosomes (GFP^+^RFP^+^ signal) (Figure S5C-D). Taken together, these findings reveal that brigatinib induces autophagic flux in CRC cells.

In order to investigate whether autophagy induction was an adaptive process in response to ER stress, we examined the expression in CRC cells of autophagy-related proteins under brigatinib treatment alone, or si*ORP8* or combined with 4-PBA. As shown in Figure 4K-L, si*ORP8* or 4-PBA obviously prevented brigatinib-induced up-regulation of autophagy-related proteins. In contrast, an early autophagy inhibitor (3-Methyladenine, 3-MA) had no obvious effect on the expression of classic ER signaling protein markers in brigatinib-treated cells (Figure S5E). These data indicate that brigatinib-induced ER stress is an upstream event of autophagy. To further investigate which pathway was involved in ER stress-induced autophagy, we prioritized the IRE1α/JNK signaling, a major pathway accounting for ER stress-mediated autophagy 36-38. As expected, brigatinib indeed activated the IRE1α/JNK signaling pathway in CRC cells (Figure S6A-C). In addition, *IRE1α* knockdown (Figure 4M) showed effective inhibition of LC3B-II accumulation. In contrast, *PERK* knockdown showed no obvious inhibition of LC3B turnover (Figure S6E). Collectively, this demonstrates that brigatinib induces autophagy via IRE1α/JNK signaling pathway in response to ER stress.

### Brigatinib induces ER-phagy in CRC cells

**Figure 5:**
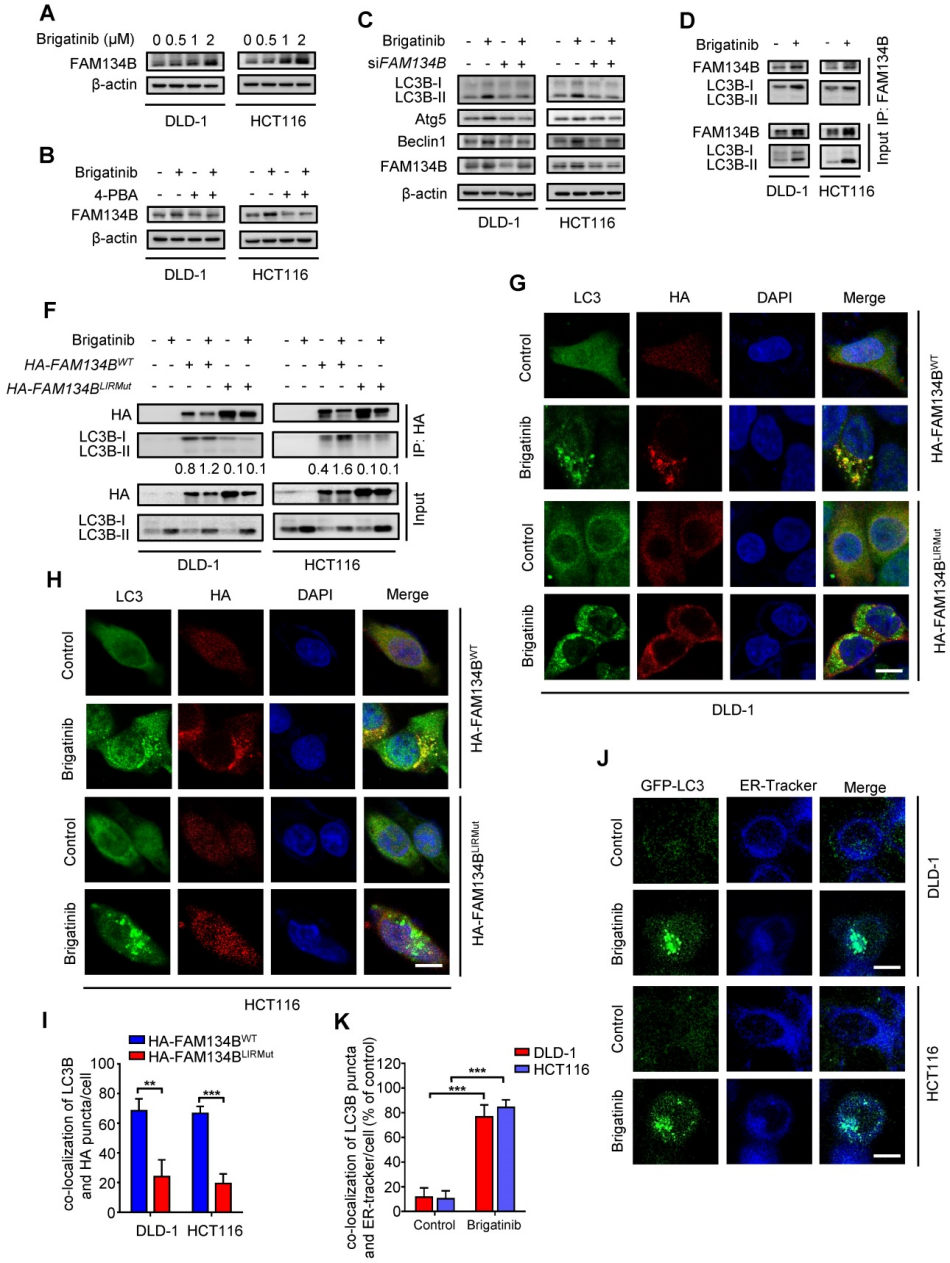
** Brigatinib induces ER-phagy in CRC cells. A,** Immunoblotting of FAM134B in CRC cells treated with the indicated concentrations of brigatinib for 24 hours. **B,** Immunoblotting of FAM134B in CRC cells treated with or without 1 μM brigatinib in the presence or absence of 2 mM 4-PBA for 24 hours. **C,** Immunoblotting of LC3B, Atg5, and Beclin 1 in CRC cells transfected with si*FAM134B* or si*Scramble* followed by treatment with or without 1 μM brigatinib for 24 hours. **D,** Interaction between FAM134B and LC3B was determined by coimmunoprecipitation assay. **E,** CRC cells transfected with either WT or LIRmut HA-FAM134B plasmids for 48 hours, followed by treatment with or without 1 µM brigatinib for 24 hours. Interaction between WT or LIRmut HA-FAM134B and LC3B was determined by coimmunoprecipitation assay.** F-H,** Immunofluorescence analysis of colocalization of LC3B with WT or LIRmut HA-FAM134B in CRC cells treated with or without 1 µM brigatinib for 12 hours. Scale bar, 10 μm. The number of colocalized LC3 puncta and WT or LIRmut HA-FAM134B (H) was quantified. **, *P* < 0.01; ***, *P* < 0.001.** I-J,** Cells were transfected with GFP-LC3 plasmid for 48 hours, followed by treatment with or without 1 µM brigatinib for 12 hours and staining with ER-Tracker Blue for 30 minutes (I). The number of colocalized GFP-LC3 puncta and ER-Tracker Blue (J) was quantified. Scale bar, 10 μm. ***, *P* < 0.001.

ER-phagy, a form of ER stress-mediated selective autophagy, modulates in ER quality control by removing dysfunctional ER 12, 39. The induction of ER-phagy requires the activation of UPR and the core autophagy machinery 9, 40. FAM134B is a common ER-anchored receptor for selective delivery of ER into autophagosomes in mammals 15, 41. As shown in Figure 5A, we observed increased expression of FAM134B after brigatinib treatment, implying that brigatinib might induce FAM134B-mediated ER-phagy. To confirm this observation, CRC cells were treated with brigatinib combined with 4-PBA (Figure 5B). As expected, inhibition of ER stress by 4-PBA reduced brigatinib-mediated up-regulation of FAM134B. Moreover, we found *FAM134B* knockdown decreased the expression of autophagy-related proteins in brigatinib-treated CRC cells (Figure 5C). The interaction between FAM134B and LC3B is a hallmark of ER-phagy 15. Using coimmunoprecipitation assay, we found increased interaction between FAM134B and LC3B following brigatinib treatment (Figure 5D-E). Next, we analyzed the colocalization of HA-tagged FAM134B with LC3B and found obvious colocalization in brigatinib-treated CRC cells (Figure 5F-H). It has been reported that specific autophagy receptors can directly interact with LC3B through LC3-interacting region (LIR) domains and FAM134B contains a conserved putative LIR motif 15, 41. Therefore, we constructed a LIR-motif-mutant FAM134B (DDFELL/AAAAAA; mutLIR) to further confirm the interaction between FAM134B and LC3B. As shown in Figure 5E-H, FAM134B^mutLIR^ rarely interacted with LC3B in brigatinib-treated CRC cells. The induction of ER-phagy was further validated by the colocalization between GFP-LC3 and ER-Tracker in response to brigatinib treatment in CRC cells (Figure 5I-J). Collectively, these results demonstrate that brigatinib induces ER-phagy, a selective form of autophagy for dysfunctional ER clearance.

### Inhibition of ER-phagy enhances the susceptibility of CRC cells to brigatinib *in vitro* and *in vivo*

**Figure 6:**
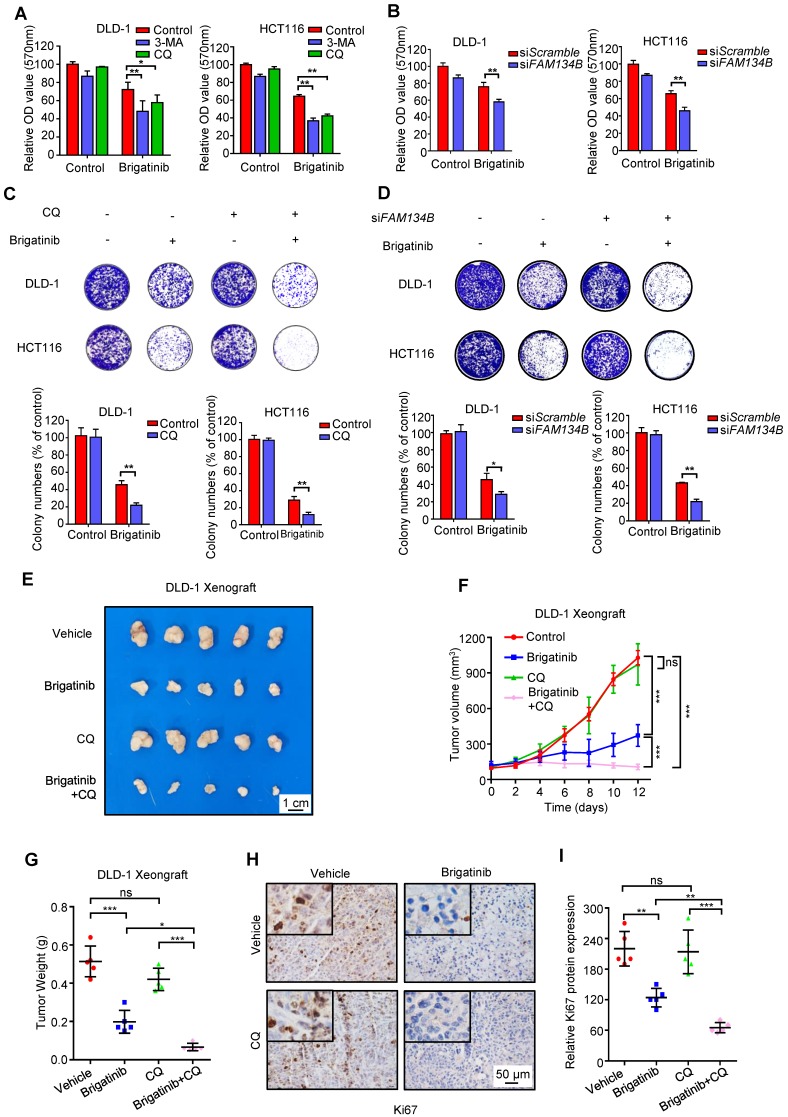
** Inhibition of ER-phagy enhances brigatinib susceptibility to CRC cells *in vitro* and *in vivo*. A,** Cell growth of CRC cells treated with or without 1 μM brigatinib in the presence or absence of 1 mM 3-MA or 10 μM CQ for 24 hours. *, *P* < 0.05; **, *P* < 0.01. **B,** Cell growth of CRC cells transfected with si*FAM134B* or si*Scramble* followed by treatment with or without 1 μM brigatinib for 24 hours. **, *P* < 0.01. **C,** Colony formation assay of CRC cells treated with or without 1 μM brigatinib in the presence or absence of 10 μM CQ. Representative images (Top) and quantification of colonies (Bottom) were shown. **, *P* < 0.01. **D,** Colony formation assay of CRC cells transfected with si*FAM134B* or si*Scramble* followed by treatment with or without 1 μM brigatinib. Representative images (Top) and quantification of colonies (Bottom) were shown. *, *P* < 0.05; **, *P* < 0.01. **E-G,** DLD-1 cells were injected subcutaneously into nude mice. When the tumor volumes reached ~100 mm^3^, mice were received vehicle or brigatinib in combination with or without CQ. Images (E) and weights (G) of isolated tumors and volumes measured at the indicated time points (F) were shown. Scale bar, 1 cm. ns, no statistical significance; *, *P* < 0.05; ***, *P* < 0.001. **H-I,** Immunohistochemical staining of Ki67 (H) and relative immunohistochemical scores (I) were shown. Scale bar, 50 μm. ns, no statistical significance; **, *P* < 0.01; ***, *P* < 0.001.

To investigate whether autophagy/ER-phagy was involved in the anti-CRC effect of brigatinib, CRC cells were treated with brigatinib combined with autophagy inhibitors, including CQ, 3-MA or Bafilomycin A1. As shown in Figure 6A and Figure S7A, CQ, 3-MA or Bafilomycin A1 led to remissive cell viability. In addition, a decrease in clonogenic survival (Figure 6C and Figure S7D) and an increase in cell toxicity (Figure S7B) were also observed following CQ or 3-MA treatment in brigatinib-treated CRC cells. Consistently, knockdown of *ATG5*, *ATG7* or *BECN1* compromised brigatinib-induced growth inhibition (Figure S7C and Figure S7E-G). These results suggest that autophagy/ER-phagy plays a protective role in brigatinib-induced growth inhibition of CRC cells. Furthermore, siRNA-mediated silencing of *FAM134B* resulted in a decrease in both cell viability and colony formation in brigatinib-treated CRC cells (Figure 6B and Figure 6D). Inhibition of autophagy/ER-phagy induced more severe apoptosis in brigatinib-treated CRC cells (Figure S7H). Together, these data suggest that autophagy/ER-phagy plays a protective role in brigatinib-treated CRC cells and inhibition of autophagy/ER-phagy results in sensitization of CRC cells to brigatinib *in vitro*.

To further explore the anti-cancer effects of brigatinib *in vivo*, a mouse xenograft model was generated by subcutaneously inoculating human CRC DLD-1 cells into nude mice. Brigatinib treatment alone demonstrated a marked reduction in the size, growth rate and weight of xenograft tumors compared with the vehicle group (Figure 6E-G). Notably, the combinatorial treatment of brigatinib with CQ showed a further decline in xenograft tumor size, growth rate and weight compared with that of brigatinib treatment alone (Figure 6E-G). Furthermore, xenograft tumors from brigatinib-treated mice showed weaker Ki67 staining in comparison with the vehicle group, and combinatorial treatment of brigatinib with CQ displayed a further reduction of Ki67 staining (Figure 6H-I). In addition, obvious apoptosis induction was observed in tumors from brigatinib-treated mice as evidenced by increased cleaved-caspase 3 levels, while combinatorial treatment displayed more robust cleaved-caspase 3 staining (Figure S7I-J). We also observed increased expression of ORP8 and LC3B by immunohistochemical staining (Figure S7K-N). Moreover, H&E staining of major organs showed no obvious toxic effects in the brigatinib, CQ or combinatorial treatment groups (Figure S8). These results indicate that CQ improves the anti-cancer effect of brigatinib by inhibiting autophagy/ER-phagy in CRC cells *in vivo*.

We further investigated whether brigatinib could show synergistic effects with 5-fluorouracil (5-FU), the first-line chemotherapeutic drug for CRC treatment, by performing the Chou-Talalay method. The combination index (CI) was calculated to evaluate the synergism (CI < 1) or antagonism (CI > 1) for each drug combination. Our results showed that 1 μM brigatinib combined with 25 μM 5-FU exhibited remunerative anti-cancer effect in CRC cells as evidenced by reduced cell growth and colony formation. In addition, the CI value of brigatinib combined with 5-FU treatment also showed the sufficient synergy (CI = 0.499 in DLD-1 cells; CI = 0.880 in HCT116 cells) (Figure S9A-C). Taken together, these results demonstrate that brigatinib can effectively sensitize CRC cells to 5-FU treatment.

## Discussion

**Figure 7:**
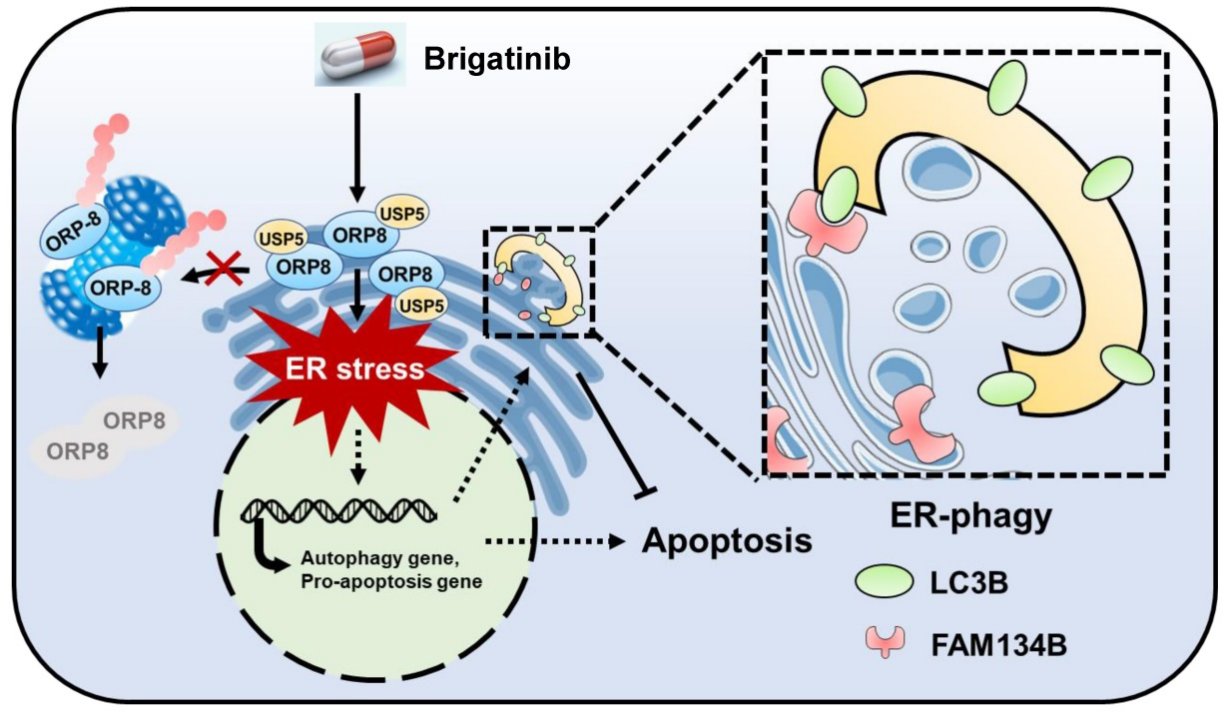
** Graph abstract: the anti-cancer mechanism of brigatinib in CRC cells.** Brigatinib enhances interaction of USP5 with ORP8 to facilitate ORP8 accumulation by attenuating ubiquitination degradation, resulting in ORP8-induced ER stress. Meanwhile, ER-phagy is stimulated to relieve ER stress in brigatinib-treated CRC cells.

Brigatinib is an ALK inhibitor used to treat ALK-positive NSCLC. In this study, we demonstrate its anti-cancer effect and the underlying mechanisms in CRC which is ALK-negative. We show that brigatinib triggers apoptosis in CRC via the induction of ORP8/USP5-mediated ER stress. In addition, we show that brigatinib activates ER stress-induced UPR to provoke both apoptosis and protective autophagy/ER-phagy (Figure 7). Combinatorial treatment with brigatinib and autophagy inhibitors potentiates the anti-cancer effect of brigatinib in CRC cells. To our knowledge, our findings describe a previously unreported ALK-independent mechanism of brigatinib in killing CRC which provides the potential alternative strategies for CRC therapy.

Drug repurposing, which has been proposed as a strategy for developing new therapies, offers a cost-effective and time-saving approach for developing promising drug for cancer therapies 42. Brigatinib is an ALK tyrosine kinase inhibitor (TKI) that was approved for the treatment of ALK-positive metastatic NSCLC in 2017 18, 19. The anti-cancer effect reported here is clearly independent of ALK, but through induction of ER stress-mediated apoptosis. Interestingly, brigatinib fails to induce ER stress in ALK-positive H3122 and H2228 cells. Our data thus underscore a novel tumor-suppressing mechanism of brigatinib. Increasing evidence supports the concept of drug repurposing for the discovery of new indications for existing drugs. For instance, metformin, which was originally used to treat diabetes in an insulin-dependent manner, showed anti-cancer effect in different cancers via an insulin-independent mechanism 43, 44. Crizotinib showed marked anti-cancer activity in ROS1-rearranged NSCLC, but it was also reported to suppress pancreatic cancer by inhibition of MET receptor tyrosine kinase 45, 46. These previous reports support our findings that brigatinib is a suitable candidate for drug repurposing and may potentially benefit a broad of cancer types.

ER-phagy, a selective autophagy process targeting the ER, requires the activation of UPR and the core autophagy machinery 9, 40. Generally, ER-phagy is a protective mechanism to maintain ER homeostasis by which excessive protein and lipid demands are fulfilled and pharmacological insults are relieved 11, 12. Intriguingly, we detected induction of ER-phagy following brigatinib-mediated ER stress. However, ER-phagy was shown to mitigate the anti-CRC activity of brigatinib. It has been reported that an unexpected pro-survival mechanism is frequently observed in ER stress-based cancer treatment. For example, flavokawain B (FKB) could be exploited as a promising anti-cancer drug candidate due to induction of ER stress, whereas provoking autophagy in FKB-treated glioblastoma (GBM) cells antagonizes ER stress-induced apoptosis 47. In addition, induction of protective autophagy has been shown to relieve ER stress in apatinib-treated CRC cells 48. These studies suggest that the blockage of pro-survival pathways emanating from the UPR potentiates ER stress-mediated cell death induced by therapeutic agents. Consistently, our study showed that combination with CQ (an autophagy inhibitor) improved the anti-CRC efficacy of brigatinib both *in vitro* and* in vivo*. This combinatorial treatment showed a comparable growth inhibition efficacy to that in ALK-positive NSCLC cells. In addition, blockage of ER-phagy by FAM134B knockdown also aggravated cell death induced by brigatinib treatment, suggesting that combinatorial treatment of brigatinib with ER-phagy inhibitor might benefit ALK-negative cancer patients. Moreover, targeting ER-phagy is more specific than targeting autophagy, which implicates notable advantage through avoiding potential side effects induced by autophagy inhibition.

Our study uncovered a novel mechanism of the USP5/ORP8 axis in the regulation of ER stress induced by brigatinib in CRC cells. USP5, a deubiquitinase, cleaves both linear and branched multi-ubiquitin polymers to influence proteasomal protein degradation 49, 50. Previous reports implied that USP5 played a crucial role in cancer therapy by regulation of various protein substrates including c-Maf and FoxM1 51, 52. Therefore, discovery of a new substrate for USP5 may be of great importance for cancer therapy. Our results suggest that ORP8 is a potential substrate of USP5. We showed that enhanced interaction of USP5 with ORP8 facilitated ORP8 accumulation by attenuating ubiquitination degradation, resulting in ORP8-induced ER stress in brigatinib-treated CRC cells. Consistent with our results, ORP8 overexpression was reported to inhibit tumor cell growth by enhancing ER stress-mediated apoptosis 29. Our findings regarding the novel link between ORP8 and USP5 help explain brigatinib-induced ORP8 accumulation and consequent ER stress in CRC cells. We then reasonably investigate whether brigatinib directly binds ORP8/USP5. It was worth mentioning that the possible interaction between brigatinib and ORP8/USP5 was preliminarily confirmed using the cellular thermal shift assay (data not shown), implying that ORP8 or USP5 might be a new target of brigatinib in CRC cells. However, the molecular basis underlying these interactions still needs further investigation.

## Conclusions

Taken together, our results indicate that brigatinib could be a promising anti-cancer drug for CRC therapy both *in vitro* and *in vivo*, and excessive ER stress might be a pivotal molecular event for tumor suppression in brigatinib-treated CRC cells. Interestingly, autophagy/ER-phagy played a protective role in response to ER stress and ameliorated the efficacy of brigatinib, whereas inhibition of autophagy/ER-phagy potentiated its anti-cancer effect. These results provided novel insights into the mechanism underlying brigatinib-induced CRC suppression, implying that brigatinib could act as a potential therapeutic agent in CRC and highlighting the possibility of autophagy/ER-phagy inhibition in optimizing cancer therapeutics.

## Supplementary Material

Supplementary figures.

## Abbreviations

3-MA: 3-methyladenine
5-FU: 5-fluorouracil
ALK: anaplastic lymphoma kinase
Baf A1: bafilomycin A1
CQ: chloroquine
CRC: colorectal cancer
DMEM: Dulbecco's Modified Eagle's Medium
DMSO: dimethyl sulfoxside
EdU: 5-ethynyl-20-deoxyuridine
ER: endoplasmic reticulum
LDH: lactate dehydrogenase
MTT: 3-(4,5-dimethylthiazol-2-yl)-2,5-diphenyltetrazolium bromide
TUNEL: terminal deoxynucleotidyl transferase-mediated nick-end labeling
UPR: unfolded protein response
