# Supplementary material for: Repurposing Brigatinib for the Treatment of Colorectal Cancer Based on Inhibition of ER-phagy
Source: Theranostics. 2019 Jul 9;9(17):4878–92. doi: 10.7150/thno.36254 (PMC6691391; doi:10.7150/thno.36254)
Supplement: Supplementary file 1 — Supplementary figures. [file thnov09p4878s1.pdf]

Figure S1

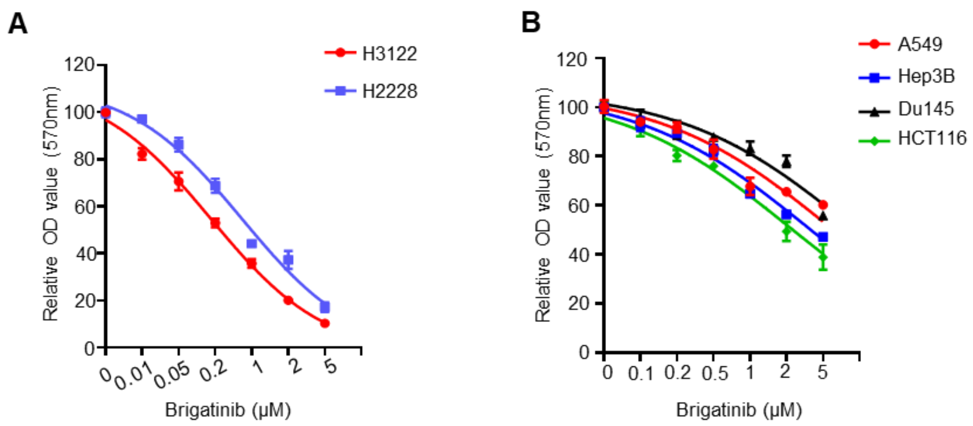

Figure S1. Brigatinib inhibits the growth of ALK-positive or ALK-negative cancer cells. A-B, Cell growth of H3122, H2228 (ALK-positive NSCLC) cells (A) and A549, Hep3B, Du145, HCT116 (ALK-negative cancer) cells (B) treated with the indicated concentrations of brigatinib for 24 hours.

Figure S2

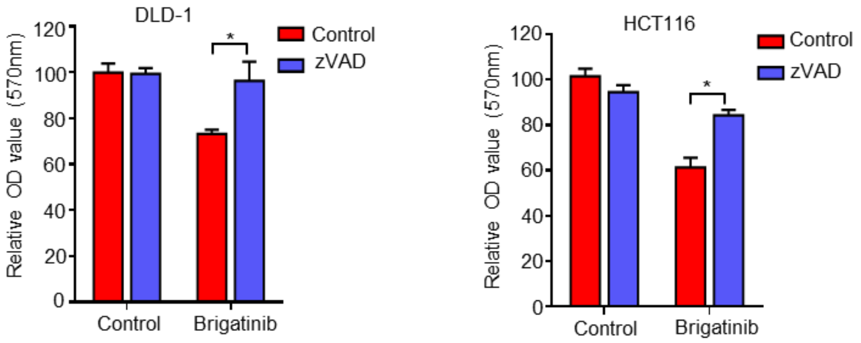

Figure S2. Brigatinib-induced apoptosis is effectively alleviated by zVAD in CRC cells. Cell growth of CRC cells treated with or without 1 μM brigatinib in the presence or absence of 40 μM zVAD-FMk (zVAD) for 24 hours. *, $P < 0.05$.

Figure S3

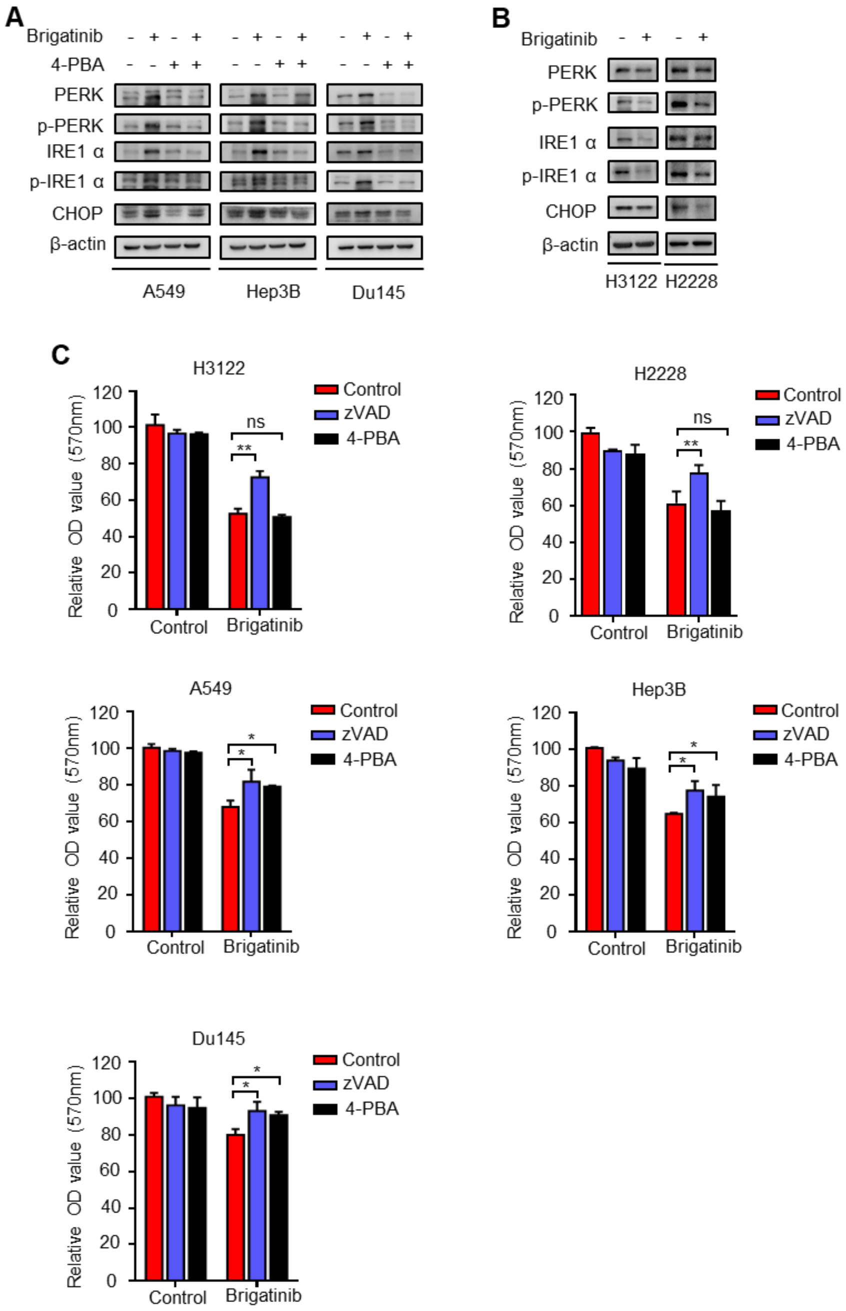

Figure S3. Brigatinib inhibits cell growth by inducing ER stress in ALK-negative but not ALK-positive cancer cells. A, Immunoblotting of total and phosphorylated PERK, IRE1α, and CHOP in A549, Hep3B and Du145 cells treated with or without 1 μM brigatinib in the presence or absence of 2 mM 4-phenylbutyrate (4-PBA) for 24 hours. **B,** Immunoblotting of total and phosphorylated PERK, IRE1α, and CHOP

treated with or without 0.2 μM brigatinib (H3122)/0.5 μM brigatinib (H2228) for 24 hours. **C,** Cell growth of H3122, H2228, A549, Hep3B and Du145 cell lines treated with or without 0.2 μM (H3122)/0.5 μM (H2228)/1 μM (A549, Hep3B and Du145) brigatinib in the presence or absence of 40 μM zVAD or 2 mM 4-PBA for 24 hours. ns, no statistical significance; *, $P < 0.05$; **, $P < 0.01$.

Figure S4

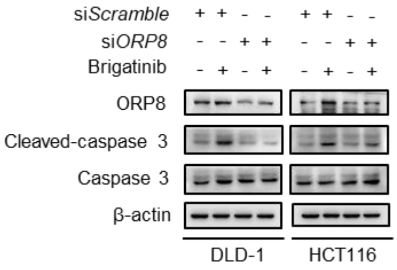

Figure S4. Brigatinib induces apoptosis via ORP8-mediated ER stress in CRC cells. Immunoblotting of total and cleaved caspase 3 and ORP8 in CRC cells transfected with si*ORP8* or si*Scramble* followed by treatment with or without 1 μM brigatinib for 24 hours.

Figure S5

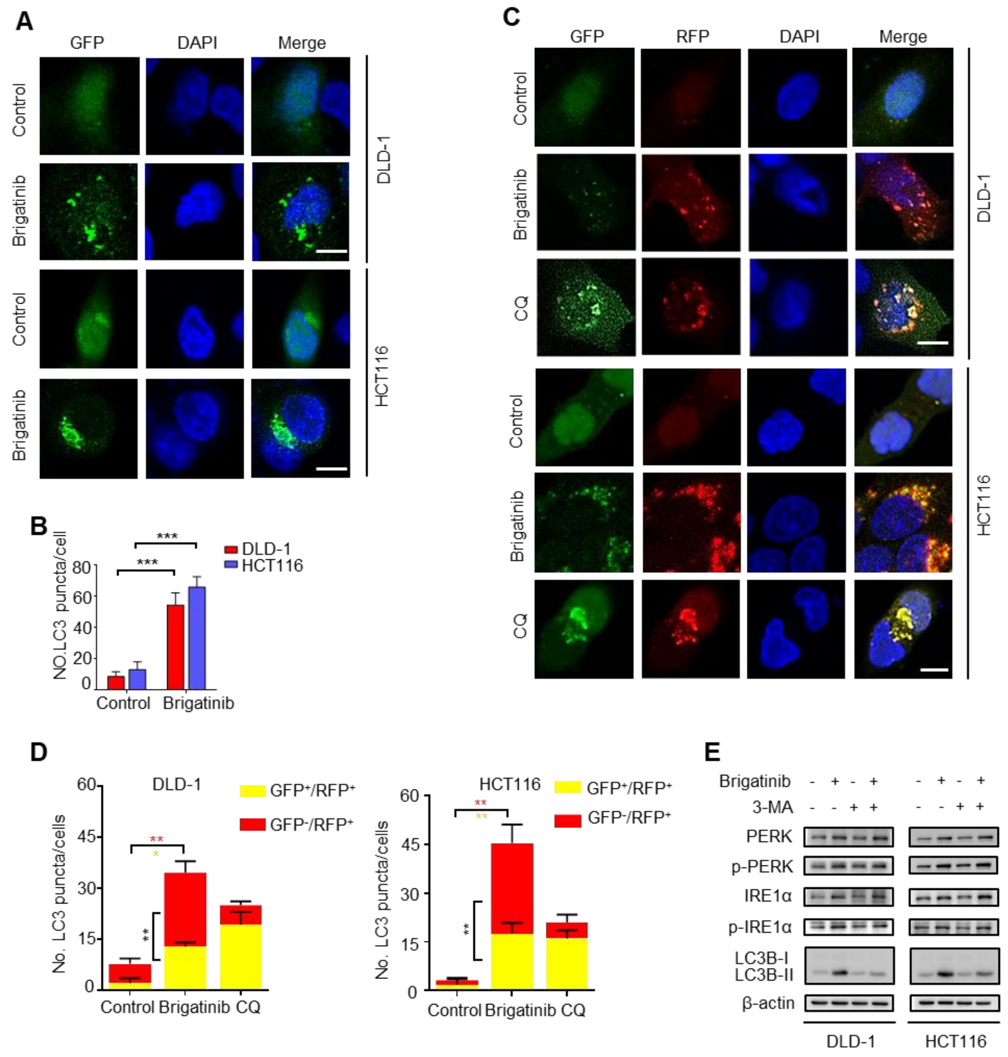

Figure S5. Brigatinib activates autophagy and promotes autophagy flux via ER stress in CRC cells. A-B, Immunofluorescence analysis (A) of CRC cells transfected with GFP-LC3 plasmids for 48 hours followed by treatment with or without 1 μM brigatinib for 12 hours. The number of LC3 puncta (B) was shown. ***, $P < 0.001$. Scale bar, 10 μm. **C-D,** CRC cells were transfected with RFP-GFP tandem fluorescent-tagged LC3 (RFP-GFP-LC3) for 48 hours followed by treatment with or without 1 μM brigatinib for 12 hours (C). The number of GFP^+/RFP^+ and GFP^-/RFP^+ (D) was quantified. *, $P < 0.05$; **, $P < 0.01$. Scale bar, 10 μm. **E,** Immunoblotting of total and

phosphorylated PERK, IRE1α, and LC3B in CRC cells treated with or without 1 μM brigatinib in the presence or absence of 1 mM 3-Methyladenine (3-MA) for 24 hours.

Figure S6

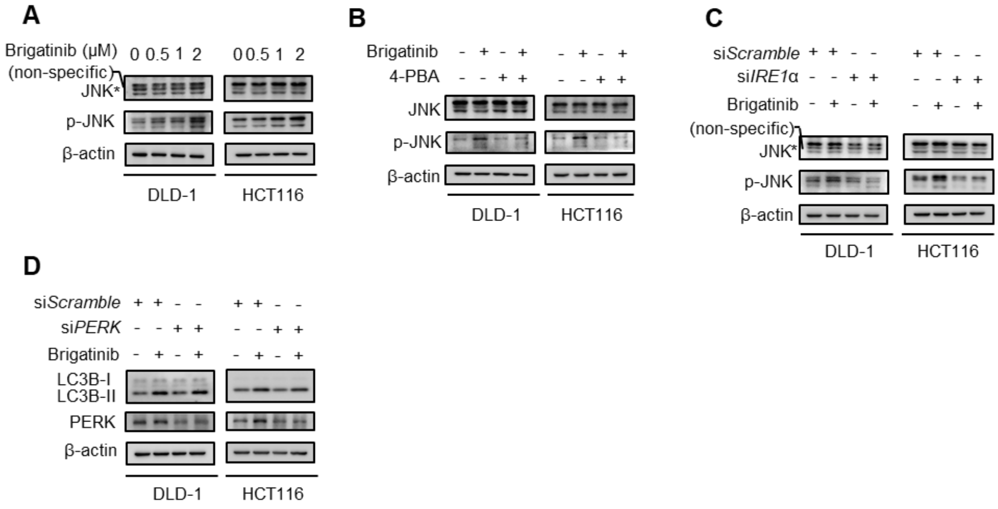

Figure S6. Brigatinib activates autophagy via IRE1α/JNK pathway in CRC cells.

A, Immunoblotting of total and phosphorylated JNK in CRC cells treated with the indicated concentrations of brigatinib for 24 hours. *, non-specific expression. **B,** Immunoblotting of total and phosphorylated JNK in CRC cells treated with or without 1 μM brigatinib in the presence or absence of 2 mM 4-PBA for 24 hours. **C,** Immunoblotting of total and phosphorylated JNK in CRC cells transfected with si*IRE1α* or si*Scramble* followed by treatment with or without 1 μM brigatinib for 24 hours. *, non-specific expression. **D,** Immunoblotting of LC3B and PERK in CRC cells transfected with si*PERK* or si*Scramble* followed by treatment with or without 1 μM brigatinib for 24 hours.

Figure S7

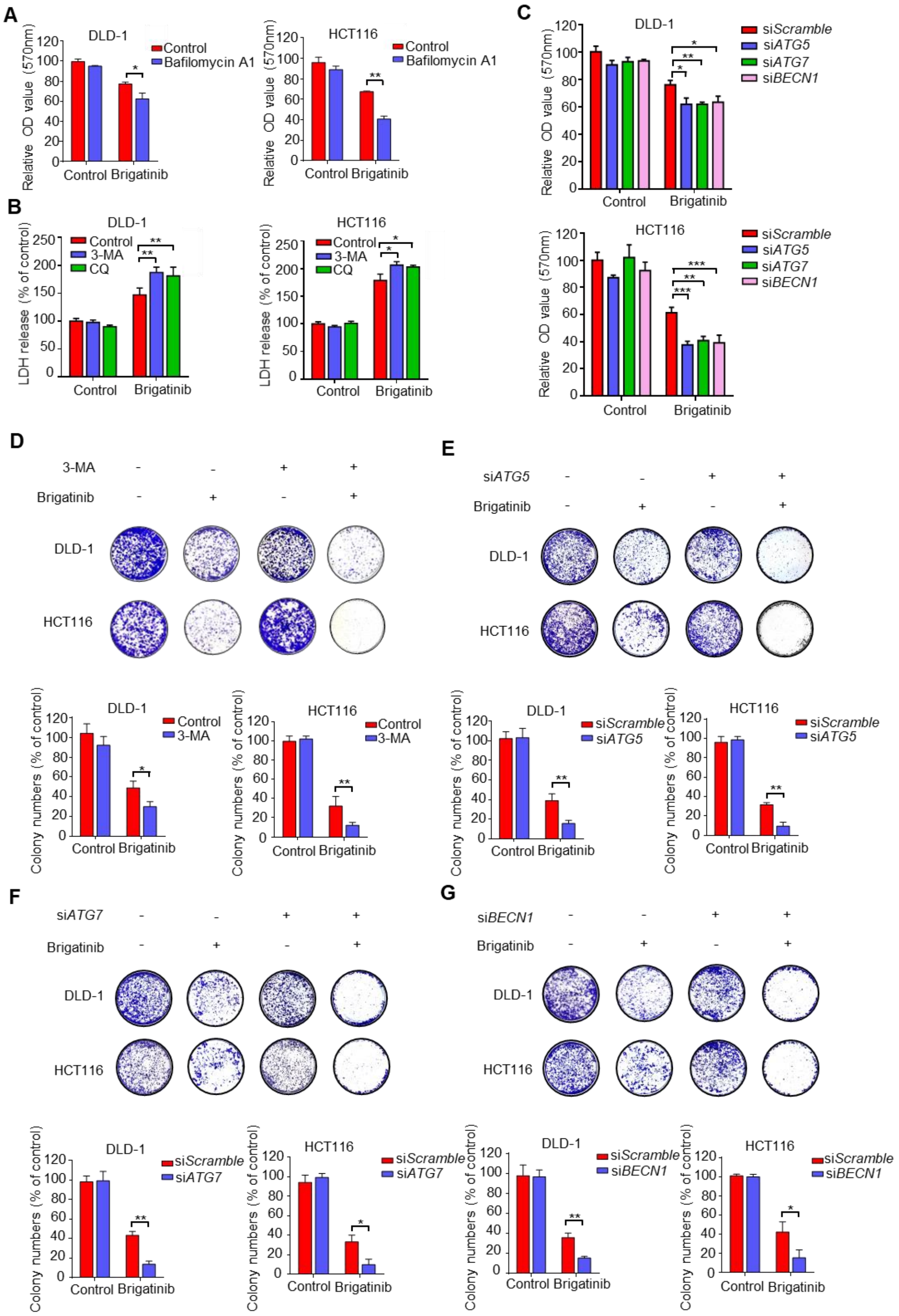

A
DLD-1
Control
Bafilomycin A1
Relative OD value (570nm)
Control Brigatinib
HCT116
B
DLD-1
Control
3-MA
CQ
LDH release (% of control)
HCT116
C
siScramble
siATG5
siATG7
siBECN1
D
3-MA
Brigatinib
E
siATG5
Colony numbers (% of control)
F
siATG7
G
siBECN1

Figure S7

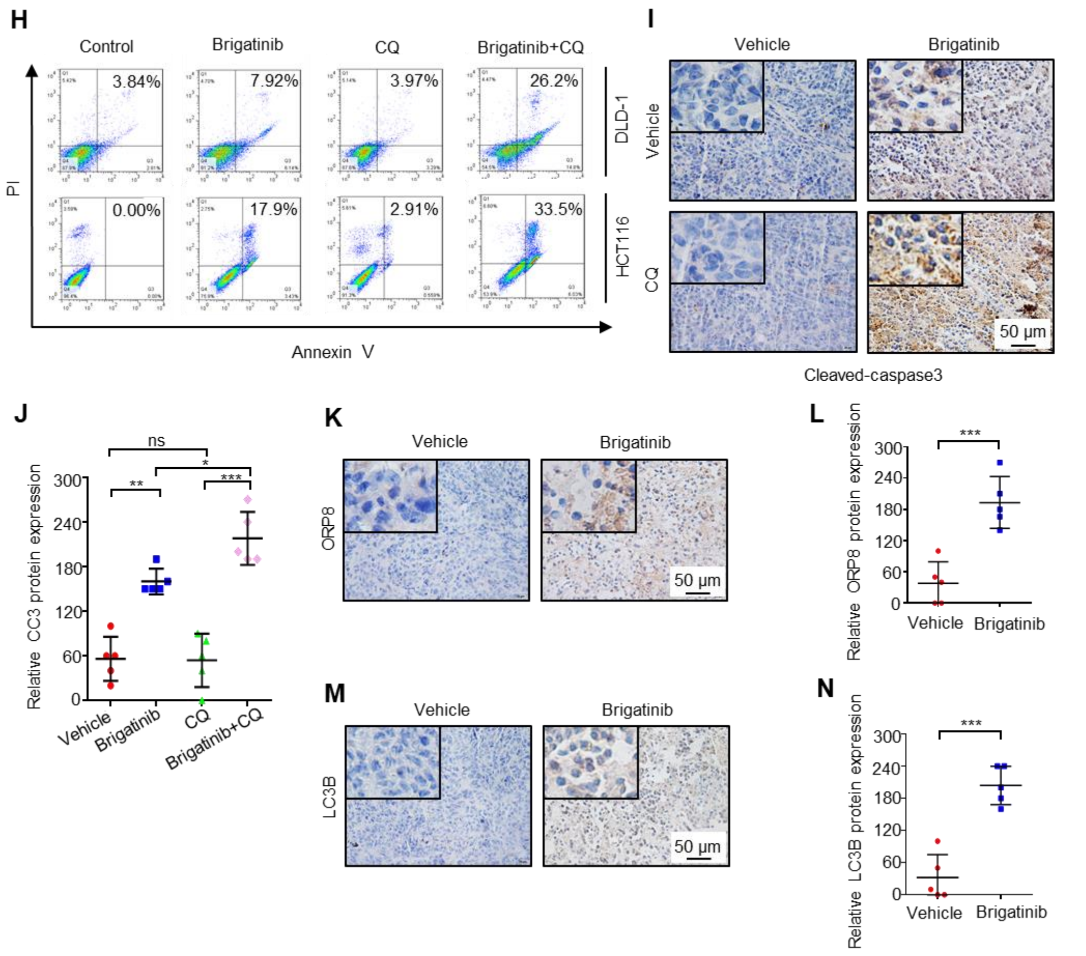

Figure S7. Inhibition of autophagy promotes growth inhibition in brigatinib-treated CRC cells *in vitro* and *in vivo*. A, Cell growth of CRC cells treated with or without 1 μM brigatinib in the presence or absence of 200 nM Bafilomycin A1 for 24 hours. *, $P < 0.05$; **, $P < 0.01$. **B,** LDH release assay of CRC cells treated with or without 1 μM brigatinib in the presence or absence of 1 mM 3-MA or 10 μM chloroquine (CQ) for 24 hours. *, $P < 0.05$; **, $P < 0.01$. **C,** Cell growth of CRC cells transfected with si*ATG5*, si*ATG7*, si*BECN1* or si*Scramble* followed by treatment with or without 1 μM brigatinib for 24 hours. *, $P < 0.05$; **, $P < 0.01$; ***, $P < 0.001$. **D,** Colony formation assay of CRC cells treated with or without 1 μM brigatinib in the presence or absence of 1 mM 3-MA. Representative images (Top) and quantification

of colonies (Bottom) were shown. *, $P < 0.05$; **, $P < 0.01$. **E-G,** Colony formation assay of CRC cells transfected with si*ATG5* (E), si*ATG7* (F), si*BECN1* (G) or si*Scramble* followed by treatment with or without 1 μM brigatinib. Representative images (Top) and quantification of colonies (Bottom) were shown. *, $P < 0.05$; **, $P < 0.01$. **H,** Flow cytometric analysis of apoptosis in CRC cells treated with or without 1 μM brigatinib in the presence or absence of 10 μM CQ for 24 hours. **I-J,** Immunohistochemical staining of cleaved-caspase 3 (CC3) (I) in tumors from vehicle or brigatinib-treated mice bearing DLD-1 subcutaneous tumor xenografts. Relative immunohistochemical scores were shown (J). Scale bar, 50 μm. ns, no statistical significance; *, $P < 0.05$; **, $P < 0.01$; ***, $P < 0.001$. **K-L**, Immunohistochemical staining of ORP8 (K) in tumors from vehicle or brigatinib-treated mice bearing DLD-1 subcutaneous tumor xenografts. Relative immunohistochemical scores were shown (L). ***, $P < 0.001$. Scale bar, 50 μm. **M-N**, Immunohistochemical staining of LC3B (M) in tumors from vehicle or brigatinib-treated mice bearing DLD-1 subcutaneous tumor xenografts. Relative immunohistochemical scores were shown (N). Scale bar, 50 μm. ***, $P < 0.001$.

Figure S8

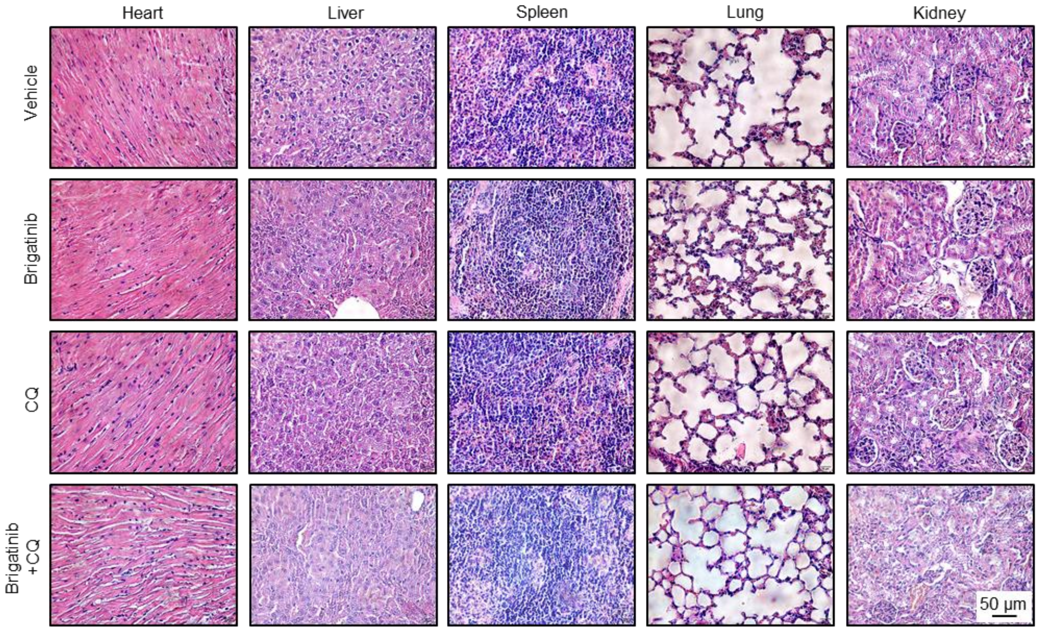

Figure S8. Brigatinib has no obvious toxicity in mice. Hematoxylin-eosin (H&E) staining of heart, liver, spleen, lung and kidney from nude mice treated vehicle or brigatinib in combination with or without CQ. Scale bar, 50 μm.

Figure S9

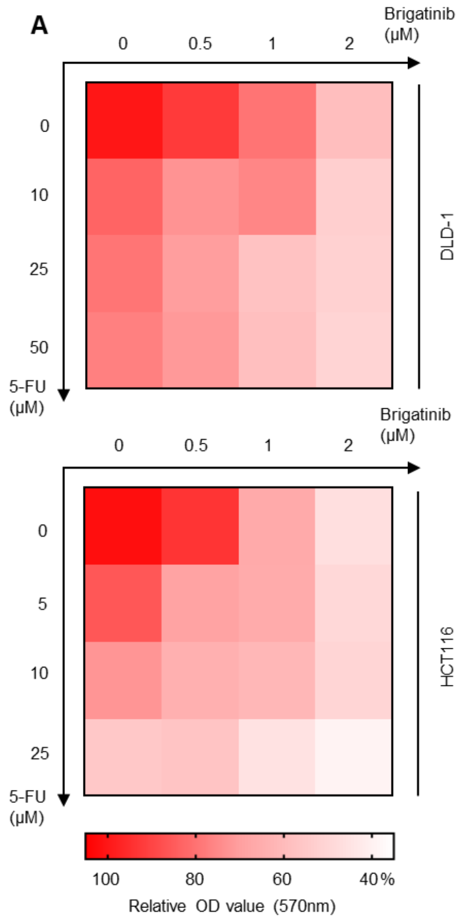

B

DLD-1

Dose (5-FU)	Dose (Brigatinib)	Effect	CI
10.0	0.5	0.273	0.465
25.0	0.5	0.297	0.481
50.0	0.5	0.286	0.686
10.0	1.0	0.243	1.003
25.0	1.0	0.427	0.499
50.0	1.0	0.414	0.534
10.0	2.0	0.475	0.860
25.0	2.0	0.483	0.845
50	2.0	0.495	0.822

HCT116

Dose (5-FU)	Dose (Brigatinib)	Effect	CI
5.0	0.5	0.311	0.790
10.0	0.5	0.359	0.861
25.0	0.5	0.435	1.038
5.0	1.0	0.344	1.134
10.0	1.0	0.384	1.162
25.0	1.0	0.545	0.880
5.0	2.0	0.51	1.207
10.0	2.0	0.496	1.357
25.0	2.0	0.609	1.104

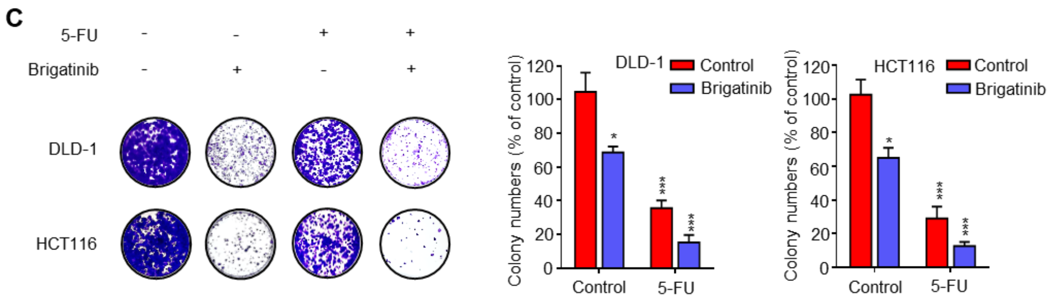

Figure S9. Brigatinib enhances the anti-cancer efficacy of 5-Fluorouracil in CRC cells. A, Cell growth of CRC cells treated with the indicated dose of brigatinib in combination with 5-FU. **B,** Combination index (CI) of the different combinations (A) of brigatinib and 5-FU were calculated to measure the level of synergism (CI < 1) or antagonism (CI > 1) using the Chou-Talalay method. **C,** Colony formation assay of CRC cells treated with or without 1 μM brigatinib in the presence or absence of 25 μM

5-FU. Representative images (Right) and quantification of colonies (Left) were shown.

*, $P < 0.05$; ***, $P < 0.001$.
